# Utilizing Theories and Evaluation in Digital Gaming Interventions to Increase Human Papillomavirus Vaccination Among Young Males: Qualitative Study

**DOI:** 10.2196/21303

**Published:** 2021-01-22

**Authors:** Gabrielle Darville, Jade Burns, Tanaka Chavanduka, Charkarra Anderson-Lewis

**Affiliations:** 1 Department of Public Health College of Health Professions Mercer University Macon, GA United States; 2 Department of Health Behavior and Biological Sciences Center for Sexuality & Health Disparities University of Michigan School of Nursing Ann Arbor, MI United States; 3 School of Health Professions College of Nursing and Health Professions The University of Southern Mississippi Hattiesburg, MS United States

**Keywords:** digital games, behavior change, theory, evaluation, game design, health care providers

## Abstract

**Background:**

Human papillomavirus (HPV) is the most common sexually transmitted infection in the United States. HPV attributes to most cancers including anal, oral, cervical, and penile. Despite infection rates in the United States, recommendations and communication campaigns have traditionally focused on females. Because of this, males lack knowledge about reasons for vaccination, the benefits of being vaccinated, and their HPV risk, overall. Gaming as a health education strategy can be beneficial as mechanism that can promote behavior change for this key demographic because of the popularity of gaming.

**Objective:**

We sought to explore the relationship between gamification and HPV vaccine uptake.

**Methods:**

Interviews were conducted with experts (n=22) in the fields of cancer prevention, sexual and reproductive health, public health, game design, technology, and health communication on how a game should be developed to increase HPV vaccination rates among males.

**Results:**

Overwhelmingly, theoretical models such as the health belief model were identified with key constructs such as self-efficacy and risk perception. Experts also suggested using intervention mapping and logic models as planning tools for health promotion interventions utilizing a digital game as a medium. In game and out of game measures were discussed as assessments for quality and impact by our expert panel.

**Conclusions:**

This study shows that interventions should focus on whether greater utilization of serious games, and the incorporation of theory and standardized methods, can encourage young men to get vaccinated and to complete the series of HPV vaccinations.

## Introduction

According to the Centers for Disease Control and Prevention, human papillomavirus (HPV) is one of the most common STIs among adolescents and young adults [1]. Approximately 14 million new cases of HPV occur in the United States each year, with nearly 80 million people estimated to be currently infected [1,2]. An equal opportunity virus affecting both men and women, HPV can be spread easily during skin-to-skin contact during anal, oral, or vaginal sex [2,3]. Routine vaccination starting in early adolescence (aged 11 or 12 years) can prevent common HPV infections and their associated diseases, such as oropharyngeal and cervical cancers [1,3,4]. In comparison to females, males are less likely to be partially or completely vaccinated [3,5,6]. While there are 3 approved vaccines for use in the United States that are covered by insurance, vaccination rates remain low among young men [1,6].

The HPV vaccine has been available to males since 2009, and in 2019, the Advisory Committee on Immunization Practices recommended catch-up vaccinations among this population, with certain special populations being immunized up to age 26 years [1,5]. Yet, recent statistics have shown that because of the lack of or delayed symptoms with HPV-associated diseases, adolescent and young adult males are at a higher risk for certain HPV strains than adolescent and young adult females [3,4,7]. To date, there is no approved HPV test for men. Furthermore, routine screening is not recommended by the Centers for Disease Control and Prevention for HPV-related diseases that may be associated with anal, penile, or oropharyngeal cancers [3]. According to one major study [5], males lacked knowledge about HPV and perceived no benefit to getting the vaccine. In this same study, they were also unaware that they could be given the vaccine and did not receive any HPV-related recommendations by their health care providers [5]. Additionally, socioeconomic and behavioral factors, including substance abuse, racial and ethnic disparities, sexuality, distrust of the health care system, stigma, and lack of access to health care have increased the spread of the STI [5,8]. With that being said, innovative strategies need to be employed to facilitate the uptake of this vaccine.

Access to digital materials has been shown to be a highly effective method of receiving, retaining, understanding, and actionizing health information [9]. The use of interactive games (also referred to as serious games) has increased in popularity among health professionals as a viable educational tool and behavior intervention strategy for addressing health disparities. Gamification is the use of digital game mechanisms in a nongaming context to engage users, motivate activities, and solve problems. Gamification can prove to be better than traditional programs and interventions because digital games provide immediate feedback when the player takes an action or selects an option within the game. Because of this feedback (negative or positive), players are able to correct their decisions or make changes to their strategy. This feedback is beneficial to the user because it allows for increased cognitive processing and experiential learning [10,11]. When designing a serious game, gaming components should be based on behavioral theories that are capable of measuring its impact and effectiveness on individual-level determinants [12]. Interactive digital interventions including health promotion games that are based in theory, such as the self-determination theory [13,14]. and dual-processing models of cognition, were effective in educating and reducing the risk of STIs including HPV [15].

While gaming has been utilized in many domains, very few have explored gaming for vaccination uptake within public health. Furthermore, of the games developed around the influenza and general vaccinations, they have been widely researched as preventive and care engagement tools. In a systematic review [16], published in 2016, that highlighted these games, none utilized behavioral change theory. Additionally, they lacked specific evaluation methodologies and long-term assessments of the games [16]. To our current knowledge, there is only one interactive game that exclusively explores improving HPV vaccination for boys and girls aged 11 to 12 years [13]. In a 2019 study [17] conducted in Norway, researchers developed and released a mobile HPV game [17]. However, this game was not theory-based and is only meant to increase awareness and knowledge of HPV. A game that is developed as a health intervention must have the ability to be appropriately evaluated and engaging [18]. In the absence of standardized evaluation methods for serious games, design principles (ie, usability, playability, and visualization) are incorporated into designing a gameplay experience that does not distract the user from the intended outcomes. This must be simultaneously balanced with meeting user expectations of a gaming experience that combines challenge, fantasy, and curiosity [19]. Recent work has begun to explore digital gaming to improve HPV and HPV-related vaccine outcomes among young men [20], but to date, little attention has been focused on designing and evaluating an evidence-based serious game targeting HPV vaccination tailored for young men.

This study seeks to explore the relationship between gamification and HPV vaccine uptake. With this approach, we will examine (1) theoretical models that would be most useful in game design to increase HPV vaccination rates in young males aged 18 to 26 years and (2) explore evaluation study designs for digital-based gaming. As an area with limited research, digital games that incorporate theory and integrate evaluation may have the potential to normalize perception of sexual health and STI prevention and to improve vaccination rates among this population [8].

## Methods

### Participants

This study was approved by the University of Florida’s institutional review board. Experts were recruited from the fields of sexual and reproductive health, cancer prevention, public health, game design, and technology and health communication to collect qualitative data. Because the topics of HPV and gaming are interdisciplinary in nature, we aimed to get at least 2 experts from each of the fields mentioned above to participate in the interviews. Besides the disciplines within which experts were employed, sampling was not done based on any other variables. A nonrandom purposive sampling approach was implemented to ensure heterogeneity with respect to expert backgrounds. Experts were initially identified based upon their publication record, word of mouth recommendations, and body of work or experience. Initially, a total 18 experts were contacted and invited to participate in the interviews. All 18 experts accepted the invitation to participate in the study. A snowball method of interviewee identification and selection was also employed to recruit additional participants. At the end of each interview, experts were asked whom else the research team should contact to participate in the study. An additional 6 experts were referred and invited to participate based on their relevant areas of expertise. One of the referred experts declined to participate.

### Procedure

The purpose of the interviews was to elicit in-depth input and recommendations to explore the current state of games for health research and practice and strategies for using games as a means to promote and improve HPV vaccination rates among college-age men. In-depth interviews were carried out with the identified experts who were contacted via email and invited to participate in the study. If the response was positive, a telephone or in-person interview was scheduled by research staff.

The interviewee was provided with a copy of the interview guide (see Textbox 1) and informed consent form prior to their interview in an effort to help them prepare. Interviews were conducted using open-ended questions. All interviews were audiorecorded, and each participant was made aware of the audiorecording when they gave their verbal or written consent to participate in the study. Interviews lasted an average of 80 minutes and were conducted by the same member of the research team in a location conducive to private conversation. Interviewees were not compensated for their time but were very willing to participate due to their interest in the purpose of the interview. Because the experts had participated in interviews before, combined with the training of interviewers, all interviews were conducted in one round and sufficient detail was provided. Interviews were carried out by the first and fourth authors who were trained in qualitative research inquiry.

**Expert interview questions guide.**

1. What’s your experience in HPV research or just HPV?
2. Have you had any experience in game development design and/or testing?
3. Would you recommend someone using a digital game to improve sexual health outcomes? And why or why not?
4. What do you think are some of the benefits of using digital games for sexual health?
5. What do you think are some of the challenges of using digital games for sexual health?
6. Do you think that college men would be receptive to using a digital game about the virus and about the vaccine? And why or why not?
7. When you think of a game, a digital game aimed at increasing in particular risk perception of the HPV virus and vaccine uptake, what health messages do you think would be most beneficial for college aged males and why?
8. What features or characters should be included in the game for college age men to increase their risk perception for the virus?
9. What features or characteristics do you think would be more instrumental for increasing risk perception?
  What game mechanics would be beneficial for increasing risk perception?
10. What features or characteristics do you think would be more instrumental for increasing HPV vaccine uptake?
  What game mechanics would be beneficial for increasing HPV vaccine uptake?
11. If you were to design a game on the vaccine itself how would you foresee that being done? Specifically the development and designing of the game?
12. What are the most relevant theories, models, or evaluation strategies for HPV vaccine programs targeting college age students?
13. How should we evaluate the game for quality and impact?
14. How would you define success in a game for health behavior change related to the HPV vaccine?
15. Are there any other advice or recommendation would you give to someone developing a game for HPV for college age men?

### Data Analysis

Interviews were transcribed verbatim by trained transcriptionists. Transcripts were organized and coded across all 22 interviews. Using grounded theory, emergent themes were identified and reported [21]. To establish reliability, 2 independent researchers individually coded each transcript. Coding was conducted in rounds and researchers coded 5% of each transcripts then met to discuss the identified codes. By the second round all differences were discussed, and a final coding scheme was established. This approach was continued until all of the transcripts were completed. After coding, the data were sorted by themes and subthemes with accompanying quotes for clarity and organization. A matrix was used to identify patterns within the data to be explored.

## Results

### Overview

The results reported in this study reflect only a portion of the data collected from the study. Specifically, results discuss the use of theory, program planning models or approaches, and evaluation strategies that should be employed in game design and development pertaining to the HPV prevention and uptake of the HPV vaccine for college-age men.

### Theories and Theoretical Constructs

The experts who were interviewed emphasized that any gaming intervention designed for males should incorporate theory. Some theories that were mentioned in the interviews included social progress theory, social cognitive theory, normative theory, psychosocial theories, elaboration likelihood model, theory of reaction, theory of reasoned action/planned behavior, lifestyle risk deduction model, theory of gender and power, transtheoretical model (stages of change), health belief model, extended parallel process model), theory of triadic influence, integrative behavioral model, diffusion of innovation, and Anderson health care utilization model. Although interviewees either worked in research, government, or academia, collectively, they all encouraged the use of behavioral theories. In particular theories that focused on increasing a male’s perception of benefits and addressing those key barriers that could prohibit HPV vaccine uptake were highly regarded. This resulted in the health belief model being highlighted frequently.

Of the behavioral theories, constructs most mentioned being instrumental to the intervention design included perceived susceptibility, risk perception, and self-efficacy (see Table 1). In fact, 17 of the 22 interviewees mentioned self-efficacy and highlighted self-efficacy as one of the predictors of behavior change. In addition to HPV vaccine self-efficacy, consideration of gaming self-efficacy was encouraged. While men of all ages are the highest gamers, gaming self-efficacy is predicated on making sure that whatever is designed is easy enough that people with that confidence voluntarily engage in gameplay and continue this behavior [22]. Apart from self-efficacy, risk perception was the second-most mentioned theoretical construct in the qualitative interviews. While risk perception is instrumental to the uptake of the game (ie, feeling though you are at risk which encourages you to play the game to learn how to reduce your risk), interviewees felt as though highlighting that risk within the game and engaging within game risk reduction activities would be most influential to end game and out of game behavior. Overall, the two most mentioned theoretical constructs (self-efficacy and risk perception), were both components of the most frequently cited theory (health belief model) in this particular study. Thus, strengthening the health belief model’s appeal as the theoretical foundation that should be used when developing a digital gaming intervention around HPV and the HPV vaccine.

**Table 1:** Theory or theoretical construct quotations.

Frames	Quotations
Health belief model	“In this age group, I think—and I’ve not done work with this age group, its speculation—but based on different behavioral change theories, you know, breaking down some of those barriers, 'Where can they get the vaccine?'; 'Is there a cost attached to it?'; 'What are the benefits to them, specifically?'; 'What are the benefits to their relationships, that they care about?'; 'What are the potential side effects to the vaccine' and being able to sort of weigh those against the majority of the benefits that they would experience”
	“To implement your program more than anything. But if you're talking, that's not, that's not really a theory so if you're really talking about actual theories, I mean I would say more the Health Belief Model. Cause I know it was developed to...to explain vaccinations”
	“Well, health belief model is the one that’s used a lot in sexual health because it looks at perceived susceptibility of a disease or other threat and then kind of cues to action and perceived benefits”
	“I mean cause like for example, you know, you could say well people may not know where to go to get the HPV vaccination. They may think that they may have to go to the health department, they may not have health insurance... it could be a situation they could get it for free like those sorts of things. So, applying each individual... concept of the Health Belief Model, I think would be, would be really good. I think there is a very tangible, applicable, applicable way to do it”
Self-efficacy	“But you know the game while it can make them behave in a certain way, which is cool, because that is one of the advantages of the game, but it can translate to reality, we only really are targeting the cognitive things, so even when I do it I’m not only playing the game but I’m potentially changing my cognition, my behavioral capability, my self-efficacy, my knowledge and maybe that’s going to change my intention of behavior”
	“That’s a lot more interesting, and from a theory perspective, I can bide with that that’s building self-efficacy because you’re engaging in the behavior, even if it’s simulated.”
	“So, you’ve, you know you’re going to have to apply methods that will boost that self-efficacy. So, you are probably going to be using things like persuasion, modelling, um successive approximation to a goal maybe. So that makes me think about simulations, when you’re thinking about the group because they could take an avatar and take them through what they think is a risk reduction behavior and see the results or not.”
Risk perception	“You know so I think, things about genital warts, probably would be much more realistic, um, you know, improving risk perception and severity, you know, that’s where a game I think will have um, a big impact, because its um, especially if it was the simulation where um, you know like that whatever plague is that they spread in World of Warcraft, similar to that, like how easy it is to be unaffected, that content takes only one time, you know colleges full of all kinds of risk behaviors as people figure out their identity and those coupled with drinking and things like that, and the fact that this is an STD does not necessarily get protection from using condoms”
	“Well I mean if it’s risk perception is one. Low levels of risk perception is one of the determinants that are negatively influencing vaccine uptake. Then I think messages and of things like you know, 'Anyone can get it,' and maybe characters that have similar attitude of, 'Yeah I’m not— that’s not going to happen to me,' and you know it spreads the misconceptions that then are addressed and I think that those kinds of things can help. Also, you know increasing information about risk, about the common-ness of the infection”

### Program Planning Models or Approaches

In addition to theory, interviewees mentioned the role and impact of using program planning tools, approaches, and models to develop a gaming intervention for males. Items that emerged during the one-on-one interviews included intervention mapping and logic models. One interviewee preferred the intervention mapping approach because this model goes step-by-step thorough how to design an intervention program, which can also be applied to the game design and development process. Intervention mapping can also be of great benefit because it can help developers think of the determinants within the target population that should be considered and help the game be more effective. Social marketing was also mentioned as a possible dissemination tool to encourage game play and promote health among males (see Table 2).

Logic models are also an instrumental tool to program and intervention development in the fields of public health, health education, and health promotion. While it is an approach that is applied more in the traditional setting, it can be easily adapted to a virtual or technological setting. As discussed by the experts, logic models can provide a snapshot view of the entire intervention. This can include the processes that should be done beforehand (prior to game design) such as formative research and usability or beta testing, the strategies (game mechanics) that should be integrated into the game, the activities that should encourage game play and then the short-, intermediate-, and long-term outcomes that we would like to see to indicate success after the intervention is done.

**Table 2:** Program planning models quotations.

Frames	Quotations identified
Intervention mapping	“So again I’m off topic, but I think intervention mapping is my recommendation because that’s what we used here and I think it covers the basis and you can use it to the degree you want to use it in terms of the depth. But it basically says find out about the problem, come up with a concept of the solution, build a prototype to that, fix it and design, and figure out how you are going to disseminate it as well.”
	”We usually, we always use an intervention map which is a-which is an approach to develop things any kind of intervention but it really helps you think through the, you know, the-the desire to change in whatever determinants and then make decisions about what are the message and the practical applications that could influence change in the determinant. So, so a systematic process for making decisions about the approach, I think, is critical.“
	“Yeah I think that from a generic perspective I would probably recommend a process such as intervention or mapping. You’ve heard about intervention mapping......Yeah, so that’s really fairly well known. It’s a pretty standard approach to develop an intervention. What it offers to this particular behavioral science and health education is it helps with determining health behavior. So, if your game is designed to encourage risk reduction behavior um, let’s say, um then you will, you will expect to delineate the specific behaviors to, delineate the specific determinants like a social determinant of those behaviors. So you are talking about self-adequacy expectations, norms, perceived norms, whatever is important for those in your particular target population and it will sort of force you to really understand the nature of the problem and, the nature of the best delivery system”
Logic models	“So, I would do that first and then once we have that, what we do in this process that I mentioned is that we developed, you know, a logical model of change. And in that logical model of change you have the behavior that you want which in this case is probably vaccine uptake and completion of the, of the theory.”

### Evaluation for Quality and Impact

As with any health promotion program or intervention, it is important to evaluate it to determine its effectiveness. However, successful evaluation relies heavily on well thought out data collection and assessment activities. Because game design can be an expensive and lengthy process, our experts advised conducting alpha or usability testing to ensure that it captures what it needs to prior to final data collection and assessment. Apart from testing the gaming mechanics and features prior to the final development stage, they also encouraged thinking about the measures that are to be collected. More importantly developers should determine if the final measure will be collected as a pre- and post gaming intervention variable external to the game; or whether there a way to use built-in features within the game to collect data points. Measures for quality and impact discussed in the interviews included feedback from the players, message reception or acceptance, dissemination to others, social impact, and increased engagement or retention within the game. However, the greatest predictors of success, highlighted by experts, for a male gaming intervention included changes in knowledge and awareness, changes in behavior, and gaming to vaccine correlation (see Table 3). Ultimately, all interviewees who participated in our study highlighted the need to conduct process, impact, and outcome evaluations.

**Table 3:** Evaluation for quality and impact outcomes quotations.

Frames	Quotations
Alpha or usability testing	“So I think, yeah I would tell people about this game or I would play this with my friends or, I believe this game is as good as other games that are related to health or how does this game compare to the games you play? Is it better or worse? More fun less fun? These are questions that we typically ask in usability but you can ask those type of questions. One of the questions I really like is 'how could we make this game better?' And the reason I like it is that it is positively oriented, its um, it’s got an altruistic style to it and it really find out what sucks in the game without them having to say it, so it’s really acknowledging the short fall of the game. So, it’s like they can say I would really change up this and blah, instead of them having to endure the sort of social desirability problem of telling you this thing sucks”
	“Usability is giving it your user, a new player and asking the same questions. You can do usability by doing that. You could do it with a survey. You can also do talk aloud usability test. You get them to play it and tell you what is going through their mind so um well, I’m going to pick the pink button because they think that you know that’s going to lead to a better result, I’m going to choose the middle door on my quest you know, why, I’m going to choose this avatar, why? We did our Asthma simulation, we found that the young males all gravitated to this older Hispanic woman who had this lighter color hair because she was just perceived as more attractive. So, you learn a bit about how you are presenting everything with that approach. So, then you may tweak it or you may think it’s ready for a trial and you can apply it. You can get enough samples, that would give you the power to check the outcome and you can write a pre-post that randomize the laboratory conditions”
	Let’s say it’s a simple card game and so now we have all these cards and all these rules, we sort of think how are we going to play it and we play it amongst ourselves and we play it till we thin kits cool and we thin kits a good one, so let’s now take it to some of our target population and see it too and check their responses. We haven’t programmed something yet, we are still working with the concept and the rules versus a table game. So that is proof of the test of the concept and it’s an acceptability test and a usability test. But, what we’re asking, what we have the opportunity to look at is, questions that are looking for evidence like, ease of use, ease of play, um, understandability, acceptability, of this a s a game, um, credibility, I think the information here is trustworthy. The perceived value um so I think the information I am getting here will help me make better decisions in the future.”
Data collection measures	“Um, so I think that part of it is super helpful, I mean, I like love self-measurement projects where its like already built in um, I mean I’m so, but, here’s something, okay. It’s actually something we are doing with google right now. It’s really cool. Um, we have all these digital ads that are like measuring behavior physicians. So we know predominantly what websites these physicians frequent, you have our ads on there, they are exposed to various kind of ads, banner ads, PSAs, maybe a 6 second pre-roll that they can continue watching or mandatory 30 second yadda yadda. And we’re actually able to use IP address chasing to determine if they have taken an action after seeing this. And um, those who have watched our 30 second video are 300 times more likely to then search around HPV vaccines and vaccinations”
	“I don’t know how technology works but I think there are ways to do that relatively easily. Um you could say on some level just, you know one measure of success is that engaging and do they play it. And the other is does it actually have the desired effects. Maybe it has undesired effects like you wouldn’t want to measure but behavior changes are often what the ultimate end goal but sometimes were harder to evaluate and practice”
	“if there’s a point in the game on where to get the vaccine you can track to see how many people get to that point. If they get to the location maybe there’s something on that end that they could verify, sort of prize or coupon or something like that. Obviously if you build in social media then you can track those analytics as well. I think you’d have a lot of data now. You can quantify that to success”
	“It just depending on what you want your audience to do. Do you want people to remember messages? So maybe you could build in a measure for recall. Send them an email in a month or something and give them a short quiz and see if they remember that stuff.”
	“Pre and post the game playing. You could do behavioral stuff and then even long-term behavioral of your same cohort”
Gaming to vaccine correlation	“And I would think, that doing the same kind of measurement, that this would have to be a planned evaluation project from the outset where we are going to enroll 200 young men or whatever the number is and we are going to have them play this game, and we are going to naturally allow them to accept or not accept the HPV vaccine, we’re going to find out what their rates were before and after that. We are going to pick HPV vaccine naïve men, so that we know how many get vaccinated or there could easily be a survey component in it too, because I think it’s more than just one person being vaccinated”
	“Like...this is not the best evaluation but like pre-your game what percent of the students or whatever vaccinated and then after. Did you change it? You could even do like demand at the clinic”
	“So, if you’re going to do this on college campus, maybe look at the pre vaccinations levels the university implements freshman orientation than game and then you look at the end of the year you look at the vaccination levels. Or the game can be more specific, questions for user have you gotten this pre vaccination? Maybe you can throw away a pop up that they can click on to get the vaccine”

## Discussion

### General

In this study, we sought to examine the theoretical models that would be the most useful in game design for increasing HPV vaccination among adolescent and young adult males. We also explored an evaluation of study design topics (for creating digital games). Our major findings included the use of the health belief model and normative theory for game design. The major constructs that we found included perceived susceptibility and risk perception. Specifically, experts in sexual health, cancer prevention, health and communication, college health and wellness, and game development all found self-efficacy to be the primary construct for predicting behavior change. Other significant findings included the use of logic models and intervention mapping as program-planning tools and for evaluating effectiveness, measuring quality and impact, and conducting usability tests, all of which were essential in the front end of game design for this population.

Digital games that incorporate theories and integrate evaluation into their design can improve HPV vaccination among young males. Similar games for health that have used theoretical foundations have shown to be successful with changing behavioral outcomes. In a systematic review [23] conducted in 2015 on the development of digital games, of the 54 games were designed for health promotion, 7 focused on sexual health outcomes. Of the 7 games, 5 incorporated theoretical constructs in the design of the game and as outcome measures, and 2 specifically measured the effects of the games on behavior. While the sample was small, effect sizes were positive and significant for knowledge, self-efficacy and behavioral intention; which has great implications for overall behavior change [23].

While the focus of this study was college-age males who were not previously vaccination at 11 to 13 years old due to varying and outdated vaccine recommendations, the implications of this study can have greater impact upon younger adolescent age males. Benefits are especially prominent if implemented and used prior to sexual activity initiation to offer full protection against HPV-related cancers. Previous research has shown that theoretical models such as the health belief model show promise as theoretical frameworks for understanding for vaccine uptake among young adult males living in urban settings [24]. Our study clarifies the broader relationships between multiple theories and constructs (including the health belief model) that can be used to measure the impact of gaming on individuals, particularly for educating this population on the importance of HPV [18].

While there are several models that can be employed, the health belief model and its varying constructs have shown to be the best choice because of its proven application. Developed in the 1950s to explain why people did or did not participate in programs to prevent disease and illness, the health belief model is one of the most known and widely applied theories [25]. Specifically, in public health, it has been proven effective in behavioral interventions focused in areas such as health screenings, immunizations, vaccines, sexually transmitted infections, and cancer prevention [25].

In order to predict behavior change, the health belief model takes into account the perceived severity of the disease or illness, susceptibility to the disease or illness, benefits and barriers to performing a new behavior, and cues to action (motivators) to changing behavior. Often, perceived severity and perceived susceptibility are collectively referred to as perceived threat or perceived risk. Self-efficacy was later added to the theory, because the importance of one’s confidence in their ability to change their individual behavior was identified as an important component to behavior change as well [26]. Therefore, when developing a digital game to increase vaccine uptake, one must take into consideration the self-efficacy of the players. Self-efficacy, perceived susceptibility to HPV, and extensive exposure to HPV-related information have been shown in previous studies to predict vaccine acceptance and uptake by young males [24,27]. Self-efficacy is an essential construct, as it regulates several cognitive processes for developing and maintaining behaviors [28]. These include confidence, control, and success with specific tasks [28]. In this case, the goal is to design a game that educates and creates an experience for an individual involving HPV vaccination and sexual health promotion using the identified theories and constructs. The aim is to develop behavioral skills that will promote consistent vaccination among this population.

This study also showed the importance of evaluating game design for quality, impact, and effectiveness. Logic models are essential because they provide an organized schematic depiction of the way theory and intervention intersect, and they can be useful for understanding how outcomes are produced for a given project [29]. Intervention mapping may also be useful in designing a game to promote HPV vaccination, as it is a systematic way of looking at a health problem, design materials, and protocols based on theory and practice [30]. Historically, beta testing a game for playability, usability, visualization, effectiveness, and quality has been essential to the evaluation of any serious game [19]. In all, these data suggest that understanding the purpose of theory and evaluation may have a profound impact on the quality of the game design and on what individuals learn about HPV, sexual health, and STI prevention.

The limitations of this study include the interviewees personal biases as qualitative researchers. Qualitative research can depend heavily on the skill of the individual researcher and is thus easily influenced by researchers’ values and opinions. [31] Another bias could have emerged through the use of purposive and snowball sampling to recruit study participants. This type of nonprobability sampling often relies on the judgement and discretion of the researcher making it subjective in nature. It is also argued that this type of sampling technique is not truly representative of the entire population [32]. However because of the interdisciplinary nature of this project and specificity of the results targeting to a specific population that the methodology employed is not as much of a limitation overall. In fact, reviewing the data with peers and experts in the field can help one maintain objectivity in the data-analysis process. Another limitation is that the qualitative data gathered here are a subset of a broader data set. The overall volume of qualitative data can affect the analysis and the interpretation of the findings [33,34].

### Future Implications

Although a large majority of interventions and programs are implemented in traditional health education settings, health care providers and nurse practitioners are becoming increasingly vital to increasing vaccination rates among adolescent and young adults [35,36]. Most often, these programs or approaches are implemented in health care settings and school-based programs. In clinical settings recommendations by health care providers are cited as the primary motivator for HPV vaccination of children by parents [37]. Therefore, there has been great discussion and emphasis on the nurse’ role as a champion in promoting the HPV vaccine especially since they interact more with families during in clinic visits [35]. Alternatively, in a 2014 study [36], it was identified that public health nurses used a variety of strategies to increase the rate of HPV vaccination in schools. These included but were not limited to providing HPV health education sessions alongside an informative package, question and answer sessions, flexible appointments, the inclusion of males in health promotion activities, and communication through a variety of media formats such as the school newsletter and website [36].

Since the COVID-19 pandemic has affected medical visits and school-based interactions (because of social distancing, stay at home orders, and governmental bans), we have seen the number of vaccinations, including HPV vaccinations, decline dramatically over a short period [38]. Coupled with the already lagging vaccination rates, this is cause for concern [39]. With the increase of telemedicine and telehealth to respond to the need of health service provision, health care providers, public health practitioners, and nurses should consider gamification activities and digital games as a way provide accurate information and initiate behaviors that would lead to HPV vaccine uptake [40].

Consistently using data reduction methods can make the concepts and relationships in the interviews more visible [34]. The limitations notwithstanding, our findings contribute to the games for health literature from an evidence-based perspective. Future research should consider using focus groups with young men to aid in identifying salient content for a serious game intervention. Creating a safe space to educate young men around safe sex practices and vaccination can help to promote discussions around use, health benefits, and concerns about the virus and vaccine.

### Conclusion

Research in this area should focus on whether greater utilization of serious games and the incorporation of theory and standardized methods can encourage young men to get vaccinated and complete the series of the HPV vaccinations. In analyzing the effectiveness of a digital game, the focus should be on change in uptake measures that include not only vaccination initiation but 3-dose completion. Moreover, individuals designing health interventions for practice should consider tailoring the design to role models and or opinion leaders within the community. By providing information and focusing on the rewards to vaccination, gaming can create a constructive learning model and make learning personalized, interactive, and fun.

## Abbreviations

HPV: human papillomavirus
STI: sexually transmitted infection
